# Infection-driven proliferative phase impairment in chronic wounds: a mechanistic framework for precision regenerative therapy

**DOI:** 10.3389/fimmu.2026.1803023

**Published:** 2026-05-14

**Authors:** Qingmei Yang, Chuyu Liu, Qi Wang, Jing An, Yulan Cai

**Affiliations:** 1 Department of Endocrinology, The Second Affiliated Hospital of Zunyi Medical University, Zunyi, Guizhou, China; 2 Zunyi Medical University, Zunyi, Guizhou, China; 3 Thyroid and Breast Surgery, The Second Affiliated Hospital of Zunyi Medical University, Zunyi, Guizhou, China; 4 Department of Endocrinology and Metabolism, Affiliated Hospital of Zunyi Medical University, Zunyi, Guizhou, China

**Keywords:** antivirulence therapy, biofilm-associated pathogenicity, chronic wounds, immunometabolic dysregulation, infection-driven proliferative phase impairment, precision regeneration, wound microbiome

## Abstract

Chronic wounds represent a major clinical challenge characterized by persistent failure of tissue repair, a phenomenon that cannot be fully explained by infection and inflammation alone. Emerging evidence indicates that wound-associated microbial communities establish stable pathogenic ecosystems that specifically disrupt the proliferative phase of healing, the critical stage responsible for cellular expansion, angiogenesis, and extracellular matrix reconstruction. Here, we propose the conceptual framework of infection-driven proliferative phase impairment (IDPPI), which describes a pathological state in chronic wounds wherein sustained microbial pathogenic activities continuously compromise host regenerative programs. We synthesize current evidence showing that coordinated virulence factor deployment, biofilm persistence, and host immune–metabolic dysregulation converge to induce proliferative arrest. They do so through direct cellular injury, suppression of repair-related signaling pathways, and disruption of cell-cycle control. This integrated pathogenic cascade ultimately locks wounds into a state of low-efficiency or arrested regeneration. Building on this mechanistic framework, we outline a sequential, targeted therapeutic paradigm encompassing three interconnected levels: targeted suppression of virulence and biofilm functions, restoration of immune–metabolic homeostasis within the wound microenvironment, and spatiotemporally controlled promotion of regeneration using responsive biomaterials and cell-free regenerative strategies. Rather than prioritizing non-selective microbial eradication, this approach emphasizes functional disarmament of pathogenic ecosystems and reactivation of host proliferative capacity. Finally, we discuss how advances in spatial multi-omics, biomimetic human-relevant models, artificial intelligence, and real-time sensing technologies can enable dynamic assessment and adaptive intervention, supporting a paradigm shift in chronic wound management from static staging toward feedback-guided (closed-loop), mechanism-informed regenerative medicine. IDPPI is presented as an integrative framework that reorders causality by placing infection-driven disruption of proliferative repair execution as the proximal failure mode.

## 1 Introduction: from persistent inflammation to infection-driven proliferative failure

Chronic wounds are commonly defined as tissue injuries that fail to progress through normal healing trajectories within an expected timeframe, typically persisting for more than one month. Their global incidence and associated disease burden continue to rise, representing a major and growing public health challenge (1, 2). Diabetic foot ulcer (DFU) exemplifies this clinical dilemma, with five-year mortality rates after major amputation exceeding 70% (3), underscoring the extremely poor long-term prognosis (2, 4).

Traditionally, chronic non-healing has been attributed to an imbalance between persistent infection and unresolved inflammation. Continuous microbial stimulation promotes excessive production of pro-inflammatory cytokines, including TNF-α and IL-1β, thereby delaying wound closure and tissue repair (5). Within this paradigm, clinical strategies have largely focused on “infection control plus inflammation suppression.” However, in many patients, wounds remain stalled or recurrent despite apparent reduction of microbial burden and inflammatory activity, indicating that persistent inflammation alone cannot fully account for chronic healing failure (6). This discrepancy exposes a critical conceptual gap in our understanding of chronic wound biology.

Recent evidence points to a more fundamental mechanism: wound-associated microbes are not merely initiators of inflammation but active disruptors of the proliferative phase of healing through multiple coordinated pathways (6). *Staphylococcus aureus*(*S. aureus*) α-toxin directly injures keratinocytes and endothelial cells, impairing re-epithelialization and angiogenesis (7, 8), while effector proteins delivered by the *Pseudomonas aeruginosa* (*P. aeruginosa*) type III secretion system induce rapid host cell death and tissue damage (9, 10). Moreover, biofilms, detected in approximately 60% of chronic wounds, form structured microbial niches whose physical and metabolic properties persistently suppress cell migration, angiogenesis, and extracellular matrix remodeling (11–13). Collectively, these observations highlight an underappreciated pathogenic principle: through coordinated virulence systems and ecological advantages, microbial communities continuously interfere with the core cellular programs required for proliferative repair, driving wounds into a state of low-efficiency or arrested regeneration.

Against this background, we propose a mechanism-informed framework termed IDPPI to explain why chronic wounds, including DFU, fail to heal despite partial control of infection. Rationale and novelty. We derived the IDPPI framework by synthesizing evidence across microbiology, immunometabolism, and wound-repair biology showing that (1) chronic wounds often exhibit persistent microbial pathogenic activity despite partial antimicrobial or anti-inflammatory control, and (2) this activity maps most consistently onto failure of proliferative repair execution (cell-cycle progression/proliferation, migration, angiogenesis, and ECM remodeling). Prior paradigms have emphasized inflammation, biofilm persistence, host deficits, or binary infection control; IDPPI reorganizes their causal ordering by positioning infection-driven blockade of the proliferative repair program as a proximal failure mode. IDPPI defines a pathological state in which sustained microbial pathogenic activities, centered on virulence factors, biofilm persistence, and host-microbe interactions, specifically compromise the proliferative repair program, encompassing cell-cycle progression, regenerative signaling, and tissue reconstruction. In this review, we integrate evidence at the interface of microbiology and tissue repair biology, focusing on three interconnected dimensions: (1) microbial direct action strategies, from ecological colonization to molecular virulence mechanisms; (2) disruption of host repair networks induced by persistent microbial activity; and (3)mechanism-guided interventions for stratified diagnosis and intervention, spanning biomarker assessment to clinical translation.

To further clarify the conceptual distinctions between IDPPI and existing paradigms, a comparative summary is provided in **Table 1**.

**Table 1:** Comparison of IDPPI with existing paradigms of chronic wound non-healing.

Paradigm	Core focus	Why wounds fail to heal	Conceptual advance introduced by IDPPI
Inflammation-centric	Persistent inflammatory signaling	Failure to resolve inflammation delays transition to repair	Specifies infection-driven proliferative repair blockade as a proximal failure mode distinct from inflammation alone
Biofilm-centric	Microbial persistence and antimicrobial tolerance	Biofilm protects pathogens and sustains chronic infection	Links biofilm-enabled persistence to specific proliferative bottlenecks (migration, angiogenesis, ECM remodeling)
Host deficit framework	Systemic and local host vulnerabilities (ischemia, neuropathy, metabolic dysfunction)	Reduced intrinsic healing capacity	Positions microbial pathogenicity as a proximal trigger that exacerbates host-side deficits
Infection-control paradigm	Presence of infection (binary: infected vs non-infected)	Ongoing infection delays healing until controlled	Reframes “infection” as a spectrum of pathogenic activities (virulence, biofilm, host–microbe interactions)
IDPPI framework	IDPPI	Coordinated pathogenic activities arrest cell-cycle progression/proliferation, migration, angiogenesis, and ECM remodeling	Enables mechanism-guided sequencing: Disarmament → Restoration → Regeneration enablement

For translational clarity, this review uses the term IDPPI-positive to denote chronic wounds in which persistent microbial damage potential plausibly remains a dominant contributor to stalled healing and coexists with failure of proliferative repair execution. Operationally, this phenotype is suggested when: (1) the wound remains non-progressing despite delivery of foundational standard-of-care measures (e.g., debridement, offloading/pressure redistribution, perfusion optimization, and metabolic control), and (2) indicators consistent with ongoing pathogenic activity are present (e.g., recurrent biofilm/PBM barrier burden, virulence-associated injury signals, or pathobiome features compatible with coordinated pathogenic programs), together with (3) clinical evidence of impaired proliferative execution (limited epithelial edge advancement, poor granulation/angiogenesis, and/or dysregulated ECM remodeling). Because validated thresholds and prospective scoring systems are not yet established, IDPPI-positive should be interpreted as a proposed operational phenotype to guide stratification and hypothesis testing rather than a definitive diagnostic label.

To align with real-world feasibility, we propose a tiered evidence approach:

Possible IDPPI-positive: clinical stagnation plus routine bedside/standard-of-care indicators suggesting ongoing pathogenic activity and impaired repair progression.

Probable IDPPI-positive: “possible” criteria plus supportive adjunct readouts (e.g., selected point-of-care/clinic-accessible assays or microbiologic signals aligned with pathogenic programs).

**Figure 1:**
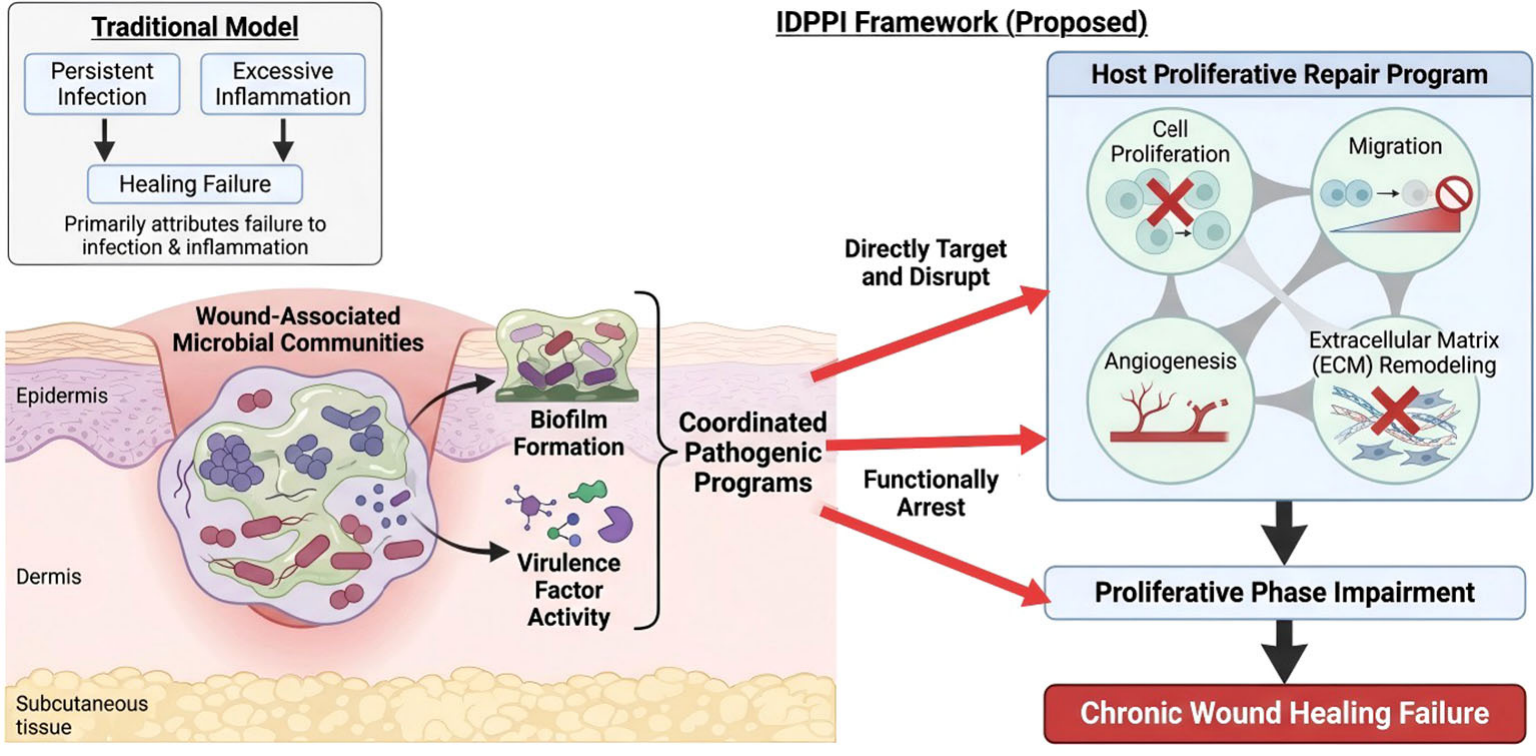
Conceptual framework of IDPPI in chronic wounds. Schematic representation of the IDPPI framework (see **Table 1** for comparison with existing paradigms). IDPPI conceptualizes infection as coordinated pathogenic activities that block proliferative repair execution, supporting a stage−specific therapeutic logic of Disarmament → Restoration → Regeneration enablement.

Research-confirmed IDPPI-positive: higher-resolution profiling demonstrating concurrent microbial pathogenic programs and suppressed host proliferative signatures (e.g., multi-omic or spatial assays), primarily in refractory wounds and research settings (**Figure 1**).

Finally, we discuss emerging technologies that may translate IDPPI into actionable wound-state assessment, including spatial multi-omics, AI-assisted analytics, and biomimetic models.

## 2 The pathogenic cooperative network: from microbial ecology to host proliferative program disruption

Pathobiome lens. In this review, we adopt the pathobiome concept to describe chronic-wound–associated microbial consortia in which community structure and function, rather than the presence of a single pathogen, drive pathology. In this view, dominant taxa, cooperative/competitive interactions, and functional outputs (e.g., virulence deployment, biofilm-enabled persistence, and immunometabolic modulation) collectively shape a disease-permissive ecosystem that can undermine repair. This framing aligns with IDPPI by specifying how the wound pathobiome sustains pathogenic activities that block proliferative repair execution even when overt inflammatory or infection signals are partially controlled (14). In chronic wounds, microbial communities are characterized by enrichment of dominant pathogens, relative ecological stability, and intensified cross-species interactions. Rather than acting as isolated insults, these communities drive a hierarchical and sustained pathogenic cascade that progressively undermines tissue repair. Following ecological colonization, dominant pathogens directly injure repair-related cells through virulence factor–mediated mechanisms, establishing the initial layer of proliferative interference. The major pathogenic species and associated virulence activities contributing to this early disruption are outlined in **Table 2**.

**Table 2:** Pathogenic drivers that initiate IDPPI.

Dominant pathogen	Virulence-associated activity	Mode of persistence in chronic wounds	Direct impact on the proliferative repair program
*Staphylococcus aureus*	Sustained virulence factor expression	Biofilm-supported functional persistence	Induces damage to proliferative repair–related cells and impairs cell-cycle progression
*Pseudomonas aeruginosa*	Type III secretion system–associated toxicity	Chronic colonization with stable virulence phenotypes	Disrupts regenerative signaling and viability of proliferative cell populations
Virulence-dominant microbial communities	Coordinated virulence activity	Formation of a stable pathogenic ecosystem	Locks wounds into a persistent state of proliferative phase impairment

Biofilm architecture further reinforces microbial persistence and immune evasion, enabling continuous pathogenic pressure within the wound microenvironment. Over time, sustained inflammation and metabolic abnormalities converge to perturb host cell-cycle regulation and regeneration-associated signaling pathways. Together, these interconnected processes progressively erode proliferative repair capacity, constituting the biological foundation of IDPPI.

### 2.1 Ecological colonization and the emergence of a pathogenic steady state

At the ecological level, IDPPI begins with the establishment of a wound pathobiome, a community state in which polymicrobial structure and function, rather than any single organism, collectively drive pathology (14). In acute wounds, microbial signals are typically transient and are followed by coordinated immune control and timely progression into proliferative repair. In chronic wounds, microbial diversity is often reduced and the community may consolidate into relatively stable, functionally pathogenic consortia that persist under selective pressures (e.g., hypoxia, necrotic burden, high glucose, repeated antimicrobial exposure) (6).

Culture-independent profiling has repeatedly identified recurrent members of these consortia, including *S. aureus* and *P. aeruginosa*, together with obligate or facultative anaerobes such as *Finegoldia magna* and *Bacteroides* spp., as well as *Candida* spp (6, 15). These taxa are frequently highlighted because they contribute complementary pathobiome functions, including adhesion/immune evasion and toxin- or protease-mediated injury (*S. aureus*), quorum-sensing–regulated virulence and metabolic versatility (*P. aeruginosa*), and adaptation to hypoxic or necrotic microenvironments (anaerobes) (6). Across cohorts, such community states have been associated with delayed healing trajectories and worse clinical outcomes, although reported prevalence and effect sizes vary with wound type, sampling depth, and analytic platform (6, 16).

The formation and maintenance of this pathogenic steady state are shaped by interspecies interactions and ecological “priority effects” (6, 17, 18). For example, early colonization by *Candida albicans* can provide biofilm-associated scaffolds that facilitate subsequent bacterial attachment (e.g., *Citrobacter freundii*) *(*18, 19). During co-colonization, *P. aeruginosa* can secrete metabolites such as 4-hydroxy-2-heptylquinoline N-oxide (HQNO), which suppresses *S. aureus* respiration and promotes small-colony variants (SCVs) with enhanced tolerance and persistence (17, 20). In parallel, host constraints typical of diabetic wounds (e.g., hyperglycemia, impaired perfusion and oxygenation) can enrich organisms adapted to metabolic stress while suppressing potentially beneficial commensals, further stabilizing community structure (6). Biofilm-associated interactions and community-level features contributing to stabilization of this pathobiome steady state are summarized in **Table 3**.

**Table 3:** Biofilm-mediated stabilization of proliferative phase impairment.

Biofilm-associated feature	Functional role in chronic wounds	Effect on host clearance	Consequence for the proliferative phase
Stable biofilm architecture	Maintains long-term pathogenic retention	Limits immune access and clearance	Prevents effective initiation of the proliferative program
Biofilm-supported persistence	Prolongs functional infection	Enhances tolerance to intervention	Delays transition from inflammation to proliferation
Community-level cooperation	Reinforces pathogenic dominance	Amplifies functional tissue injury	Causes sustained impairment of proliferative execution

Importantly, this state is not merely a byproduct of impaired healing; it can actively sustain coordinated pathogenic activities (virulence deployment, biofilm-enabled persistence, and immunometabolic modulation), thereby initiating and maintaining the upstream cascade of IDPPI through disruption of proliferative repair execution (6).

### 2.2 Direct microbial injury: virulence-driven disruption of cellular and structural repair modules

Following consolidation of a pathobiome-like steady state, dominant wound pathobiome members deploy diverse virulence effectors that directly target essential cellular and structural components of tissue repair. These virulence outputs, which are core pathobiome functions, converge on compromising cell viability, extracellular matrix (ECM) integrity, immune surveillance, and epithelial barrier function, thereby undermining prerequisites for effective execution of the proliferative repair program (6).

#### 2.2.1 Disruption of cellular viability and membrane integrity

*S. aureus* α-toxin is a prototypical pore-forming toxin that binds the ADAM10 receptor on host cell membranes and oligomerizes to form transmembrane pores. This process disrupts ionic homeostasis, depletes intracellular ATP, and accelerates injury to keratinocytes and endothelial cells, thereby impairing re-epithelialization and angiogenesis (7, 8, 21). Among these, ExoU, a potent phospholipase A2, rapidly compromises membrane integrity and induces necrotic cell death. In diabetic wounds, ExoU-mediated cytotoxicity has been linked to greater tissue injury and can contribute to damage even before mature biofilm structures are established (9, 10, 22). Collectively, these cytotoxic insults directly reduce the pool of proliferative repair–competent cells.

#### 2.2.2 Extracellular matrix degradation and immune evasion

Protease-mediated effects represent another critical mode of pathogenic interference. Pathogen-derived proteases simultaneously degrade tissue scaffolding and neutralize host defense mechanisms. For example, *S. aureus* V8 protease degrades immunoglobulins and complement components, weakening opsonization and immune clearance (23). *Finegoldia magna* SufA protease targets type IV and V collagen as well as fibrinogen, disrupts extracellular matrix integrity, and inactivates the antimicrobial peptide LL-37, thereby facilitating multi-species colonization and persistence (24, 25). These processes dismantle the structural framework required for cell migration and matrix remodeling while further suppressing local immune control.

#### 2.2.3 Adhesion, internalization, and intracellular persistence

Microbial surface proteins, including the MSCRAMMs family expressed by *S. aureus*, mediate firm adhesion to extracellular matrix components, supporting stable colonization within the wound bed. In certain contexts, these interactions trigger host cell internalization, allowing pathogens to persist intracellularly and evade immune surveillance (6). Concurrent degradation of extracellular matrix and immune effectors by proteases such as V8 and SufA further diminishes local defense capacity, reinforcing chronic infection and sustained proliferative inhibition (23–25).

#### 2.2.4 Morphological transition and invasive fungal injury

Fungal pathogens also contribute to direct tissue damage through dynamic morphological transitions. *Candida albicans* switches from a yeast form to an invasive hyphal phenotype in response to environmental cues such as elevated glucose levels and acidic pH. Hyphal structures penetrate epithelial barriers and secrete aspartyl proteases, including Sap2 and Sap5, which degrade basement membrane components and weaken tissue integrity (6, 26), further compromising epithelial continuity and repair progression.

The major host cellular processes and regulatory pathways functionally disrupted by these virulence-driven injuries are summarized in **Table 4**.

**Table 4:** Host regenerative programs targeted by infection-driven proliferative arrest.

Host regulatory system	Infection-driven disruption	Physiological role in wound healing	Contribution to proliferative phase arrest
Cell-cycle regulation	Induction of cell-cycle arrest	Expansion of proliferative repair–related cells	Insufficient cellular proliferation
Regenerative signaling networks	Signal interference and dysregulation	Coordination of migration and tissue reconstruction	Failure to execute the proliferative program
Angiogenic signaling	Chronic inflammatory suppression	Support of metabolic demand during proliferation	Inadequate vascular support for regeneration
Immunometabolic regulation	Sustained inflammatory–metabolic imbalance	Resolution of inflammation and phase transition	Persistent blockade of proliferative progression

**Figure 2:**
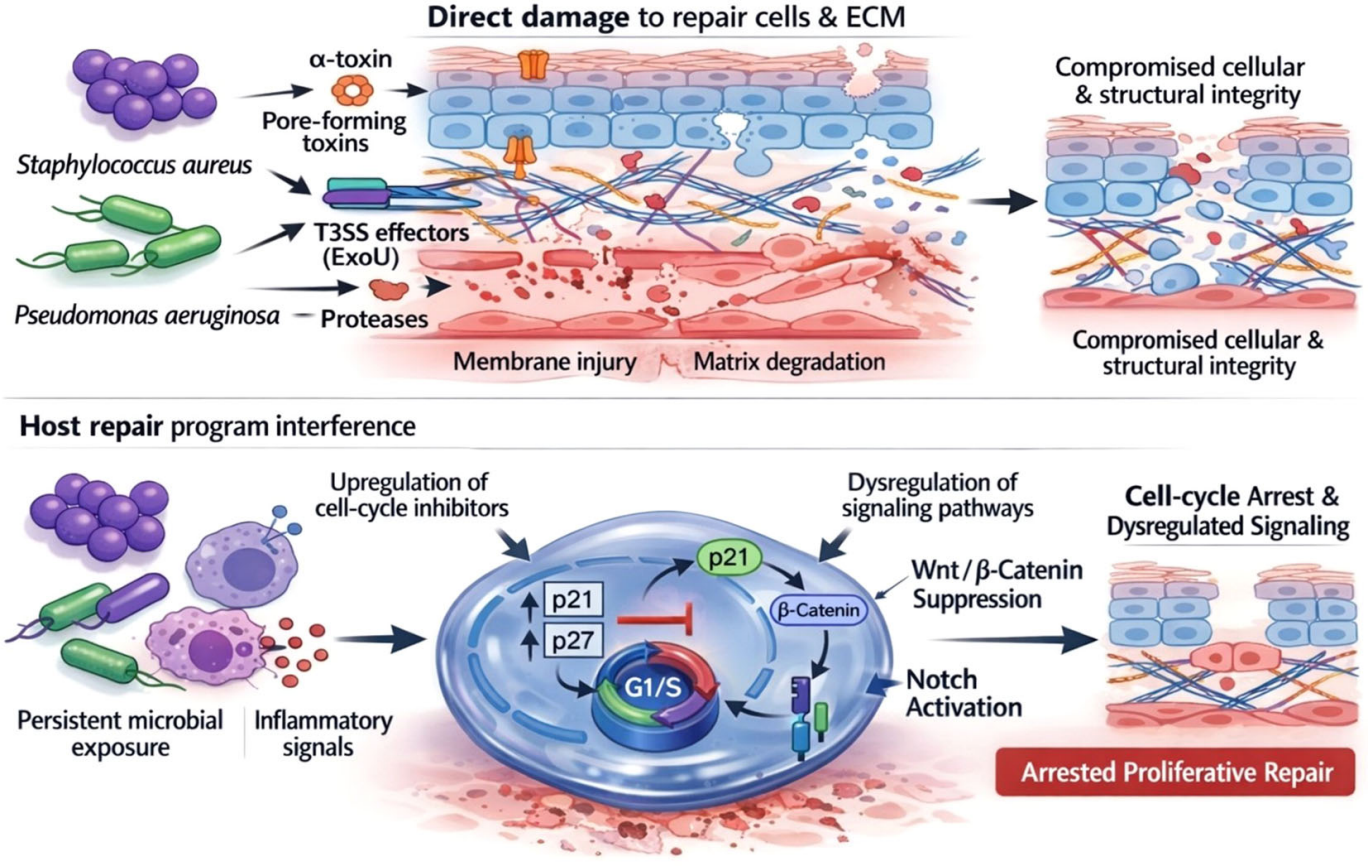
Multi-level microbial interference with host proliferative repair programs. Schematic overview illustrating how wound-associated microbial communities impair tissue regeneration through coordinated, multi-level pathogenic mechanisms. Upper panel (Direct microbial injury): Dominant pathogens, including *S. aureus* and *P. aeruginosa*, deploy virulence factors, such as pore-forming toxins (e.g., α-toxin), type III secretion system (T3SS) effectors (e.g., ExoU), and extracellular proteases, to directly damage essential repair-related cells (keratinocytes and endothelial cells) and degrade extracellular matrix components, thereby compromising the cellular and structural repair modules required for tissue regeneration (7–10). Lower panel (Host repair program interference): Persistent microbial exposure and associated inflammatory signaling further disrupt intracellular repair programs, characterized by upregulation of cell-cycle inhibitors (e.g., p21 and p27) and dysregulation of key pro-regenerative signaling pathways, including suppression of Wnt/β-catenin signaling and aberrant activation of Notch signaling. These alterations collectively impose sustained proliferative arrest and diminish regenerative output, reinforcing IDPPI in chronic wounds (5, 63, 122).

Together, these coordinated microbial insults impose persistent cytotoxic and cytostatic pressure on repair-related cells, progressively eroding cellular reserves and tissue architecture (**Figure 2**). At the systems level, this direct microbial injury constitutes a critical upstream layer of IDPPI, mechanistically linking microbial pathogenicity to sustained proliferative arrest and stalled regeneration in chronic wounds (6).

#### 2.2.5 Volatile metabolites and redox-active factors: additional infection outputs that shape pathology and monitoring

Beyond canonical toxins, proteases, and biofilm-mediated barriers, wound pathobiomes generate volatile organic compounds (VOCs) and redox-active metabolites that can both reflect and reinforce pathogenic states. VOCs are products of microbial metabolism and can encode species-/community-specific signatures with potential diagnostic value. A systematic review of non-healing surgical wounds summarizes early evidence that VOC profiling (via GC–MS and electronic noses) can associate with causative bacterial species and may support monitoring of treatment effects (27). This motivates wound “volatilomics” as a non-invasive adjunct for infection assessment.

Human skin–relevant biofilm models further support this concept: biofilms formed by wound pathogens (including *S. aureus* and *P. aeruginosa*) produce distinct VOC profiles *in vitro* and in human ex vivo cutaneous wound substrates, and selected VOCs can correlate with biofilm metabolic activity/biomass, supporting VOCs as clinically translatable microbial-function readouts rather than nonspecific malodor alone (28).

Recent analytical approaches also enable near–real-time discrimination of wound-relevant biofilm pathogens based on volatile metabolite patterns (e.g., SIFT–MS with multivariate analysis), reinforcing the feasibility of rapid VOC-informed stratification in wound infection contexts (29).

In parallel, ROS/oxidative stress represent anerappreciated axis through which infection can impair proliferative execution. Physiologic ROS are required for antimicrobial defense and signaling; however, dysregulated and persistent ROS can contribute to chronic-wound pathogenesis by amplifying tissue damage and perturbing repair-cell signaling programs (30).

Importantly, microbial virulence metabolites can directly induce sustained oxidative stress in host cells. For example, *P. aeruginosa* pyocyanin has been shown to inhibit wound repair *in vitro* in a concentration-dependent manner and to promote premature senescence phenotypes linked to oxidative stress and stress-kinase signaling (e.g., p38 MAPK), providing a concrete mechanistic route by which infection-associated redox factors can lock repair cells into non-proliferative states (31).

### 2.3 Biofilms as pathogenic barrier modules: multi-level obstruction of proliferative repair

The persistence of virulence factor–mediated injury in chronic wounds is frequently supported by biofilm growth. Biofilms are structured microbial communities embedded in an extracellular polymeric substance (EPS) matrix that provides both mechanical stability and functional advantages to resident microbes. In a microscopy-based comparison frequently cited in the wound-biofilm field, biofilm was observed in 30/50 chronic wound specimens (~60%) versus 1/16 acute wound specimens (~6%), consistent with preferential enrichment of biofilm-associated community states in chronic non-healing wounds (11). Rather than being a passive consequence of infection, biofilms can act as active barrier-forming components of the wound pathobiome that stabilize IDPPI by sustaining microbial activity and reinforcing host repair failure (6).

Here we define pathogenic barrier modules (PBMs) as functionally distinct, biofilm-enabled barrier units. Through PBMs, wound microbial communities achieve two effects (1): resist clearance and antimicrobial penetration; (2) constrain the host’s access to proliferative-phase resources and signals, such as oxygen, nutrients, growth factor gradients, and a permissive immune tone. Biofilm-mediated protection and immune evasion are well established. The added value of the PBM framing is that it maps barrier functions to specific proliferative bottlenecks (cell-cycle progression/proliferation, migration, angiogenesis, and ECM remodeling) and supports mechanism-decomposable targeting, allowing interventions to be selected based on the dominant barrier function rather than persistence alone (6, 32).

The pathological impact of biofilms on wound healing can be broadly organized into three interrelated barrier mechanisms (6, 12, 13).

#### 2.3.1 Physical–diffusional barrier

The EPS matrix, comprising polysaccharides, proteins/amyloids, lipids, membrane vesicles, and extracellular DNA (eDNA), creates a viscoelastic scaffold that can restrict migration of key repair cells (e.g., keratinocytes and fibroblasts) and limit penetration of oxygen, nutrients, and antimicrobials into deeper layers (13, 33, 34). Microbes within these protected niches often exhibit reduced growth and low metabolic activity (including persister-like phenotypes), decreasing antibiotic killing and dampening immune effector efficacy, thereby prolonging persistence and delaying clearance (13, 33, 34).

#### 2.3.2 Metabolic microenvironmental barrier

Metabolic activity within biofilms profoundly reshapes the local wound microenvironment. Gradients of hypoxia, acidity, and nutrient depletion arise from microbial respiration and fermentation, suppressing mitochondrial function and inhibiting proliferation of adjacent host cells. In parallel, microbial metabolites, including organic acids and ammonia, directly damage surrounding tissue and interfere with migration, differentiation, and functional maturation of repair cells. These microenvironmental constraints further destabilize execution of the proliferative repair program (6, 12, 13).

#### 2.3.3 Immunological barrier

Biofilm-associated EPS masks pathogen-associated molecular patterns (PAMPs), reducing recognition by innate immune receptors. In addition, biofilm-resident microbes secrete immunomodulatory factors that actively impair host defense. For example, rhamnolipids produced by *P. aeruginosa* disrupt neutrophil membranes and can induce neutrophil death/dysfunction (13, 35, 36), while other secreted products skew macrophage polarization toward dysfunctional states with diminished clearance capacity. Together, these effects blunt immune surveillance and perpetuate unresolved inflammation within the wound (13).

**Figure 3:**
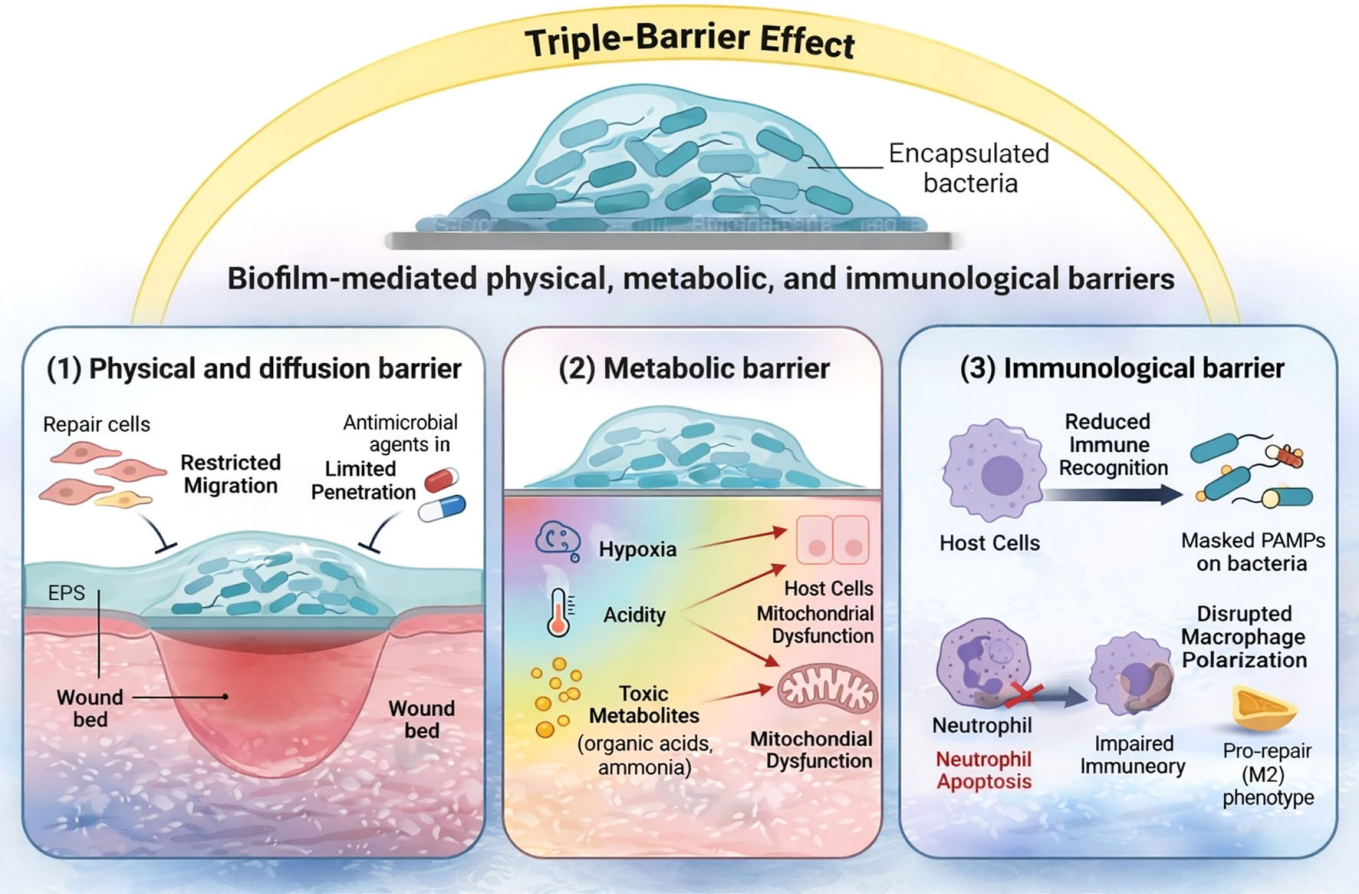
Biofilm-mediated physical, metabolic, and immunological barriers sustaining IDPPI. Schematic overview illustrating how biofilms function as structured pathogenic barrier modules that persistently impede wound healing through a coordinated triple-barrier effect. (1) Physical–diffusional barrier: The dense extracellular polymeric substance (EPS) matrix acts as a molecular sieve, physically restricting migration of repair cells and limiting penetration of antimicrobial agents, thereby preventing effective initiation of the proliferative repair program (11, 13, 34, 123). (2) Metabolic barrier: Biofilm-associated metabolic activity generates a hostile local microenvironment characterized by hypoxia, acidity, and accumulation of toxic metabolites (e.g., organic acids and ammonia), which suppress mitochondrial function and compromise viability of adjacent host cells, further destabilizing proliferative execution (12, 13). (3) Immunological barrier: EPS masks pathogen-associated molecular patterns (PAMPs), reducing immune recognition, while biofilm-derived secreted factors impair neutrophil function and disrupt macrophage polarization toward a pro-repair phenotype, collectively leading to impaired immune clearance and persistent inflammation (13, 64).

Collectively, these physical, metabolic, and immunological barriers enable persistent infection and promote transition to chronic non-healing states (**Figure 3**). Biofilms are detected at significantly higher frequencies in chronic wounds than in acute wounds and are strongly associated with prolonged healing stagnation (11, 34). Importantly, these features position biofilms as a critical and actionable pathogenic node, distinct from bacterial burden alone, and highlight their central role in maintaining IDPPI, thereby representing a primary target for mechanism-guided interventions aimed at restoring effective proliferative repair (6, 11).

##### 2.3.3.1 Pathogenic barrier modules: operational definition

A PBM is defined as a biofilm-enabled barrier unit that meets all of the following criteria:

1. Structural persistence: a spatially organized microbial community embedded in EPS with measurable stability over time.
2. Barrier function: demonstrable obstruction of at least one domain(a) diffusional/physical transport, (b) chemical/metabolic conditioning, or (c) immune/inflammatory clearance.
3. Repair linkage: mechanism-informed evidence that the barrier function constrains one or more proliferative-phase processes (proliferation, migration, angiogenesis, ECM remodeling).
4. Actionability: presence of candidate disruptors (e.g., debridement, antibiofilm agents, antivirulence strategies, immune–metabolic) that can be mapped to the dominant barrier functions.

### 2.4 Host proliferative program interference: execution failure of cell-cycle control and regenerative signaling

At the convergent downstream layer of IDPPI, sustained wound pathobiome activity (PAMP-rich microbial products, toxins/proteases, and biofilm-conditioned metabolites) combines with a chronic inflammatory/oxidative milieu to rewire nuclear programs that normally drive proliferative repair execution. This layer represents the integration point at which persistent microbial pathogenic activity is translated into durable failure of host proliferative execution (6).

#### 2.4.1 Cell-cycle arrest and proliferative paralysis

Multiple lines of evidence indicate that repair-competent keratinocytes and fibroblasts in non-healing contexts can exhibit cell-cycle arrest with reduced DNA synthesis and proliferative capacity. For example, primary fibroblasts derived from chronic wounds display G0/G1 arrest and reduced proliferation compared with fibroblasts from acute wounds (37). Mechanistically, chronic exposure to microbe-associated stimuli and inflammatory mediators can activate stress-response signaling (e.g., NF-κB/MAPK) that elevates cyclin-dependent kinase inhibitors (CDKis) such as p21 and p27, thereby suppressing CDK–cyclin activity and arresting cells at key checkpoints (G1/S and/or G2/M) (6, 38). Consistent with a causal role for p21-dependent braking in delaying the proliferative phase, increased nuclear p21 in activated wound fibroblasts has been reported to delay the onset of the proliferation phase, and local transient p21 suppression (e.g.,siRNA) can improve delayed healing in aged models (39). More recently, selective clearance of p21 high cells has been shown to accelerate cutaneous wound closure, supporting the idea that a p21-dominant arrest program can become rate-limiting for repair execution (40). Importantly, these observations help explain why reducing microbial burden alone may be insufficient in some settings: once a CDKi-high, stress-adapted program is established, repair cells may remain in a low-proliferative, senescence-like state (marked by sustained cell-cycle braking and altered transcriptional outputs) even as microbial load fluctuates (38, 40).

#### 2.4.2 Dysregulation of regenerative signaling networks

In parallel with cell-cycle arrest, chronic wound environments can show imbalanced regenerative signaling networks that coordinate proliferation, migration, lineage commitment, and matrix reconstruction. In diabetic wounds, dysregulated Wnt/β-catenin signaling has been widely discussed as a contributor to impaired proliferative responses and defective tissue reconstruction (41). Notch signaling can also become maladaptive in diabetic wounds: Notch1 activity has been reported to be elevated in fibroblasts from diabetic wounds and to suppress fibroblast growth, migration, and myofibroblast differentiation, with accompanying delays in wound repair/angiogenesis in relevant models (42). Beyond host metabolic/inflammatory drivers, microbial effectors can intersect these pathways more directly, for instance, *S. aureus* α-toxin (Hla), via its ADAM10-dependent mechanism, has been shown to activate Notch signaling in vascular cells, providing a concrete route by which microbe-derived toxins can perturb regenerative signaling modules relevant to angiogenesis and repair (43). In addition, PRR signaling has been implicated as a brake on keratinocyte proliferative behavior (e.g., TLR4-linked regulation), offering another mechanistic interface through which PAMP-rich wound microenvironments can constrain proliferative execution (44).

Together, these data support a model in which wound pathobiome activity and host inflammatory/metabolic constraints converge to enforce (1) CDKi-driven cell-cycle arrest and (2) mis-tuned regenerative signaling, thereby producing durable failure of proliferative repair execution.

Caveats on evidence strength. We acknowledge that direct causal evidence linking specific microbial community states to proliferative arrest in human chronic wounds remains limited. Most mechanistic insights derive from preclinical models (e.g., diabetic mouse wounds, *in vitro* co vitroca systems) and associative human studies (e.g., microbiome profiling, biomarker correlations). The IDPPI framework is therefore presented as an evidencedonshored organizing framework to guide hypothesis generation and translational research, rather than a validated causal model.

Collectively, chronic wound–associated microbial communities impair proliferative repair through a hierarchical pathogenic cascade encompassing ecological consolidation, virulence factor–mediated injury, biofilm/PBM stabilization, and ultimately disruption of host cell-cycle control and regenerative signaling. These interconnected mechanisms constitute the mechanism-informed core of IDPPI (6).

## 3 Framework-informed targeted therapy: sequential reactivation of proliferative repair

**Figure 4:**
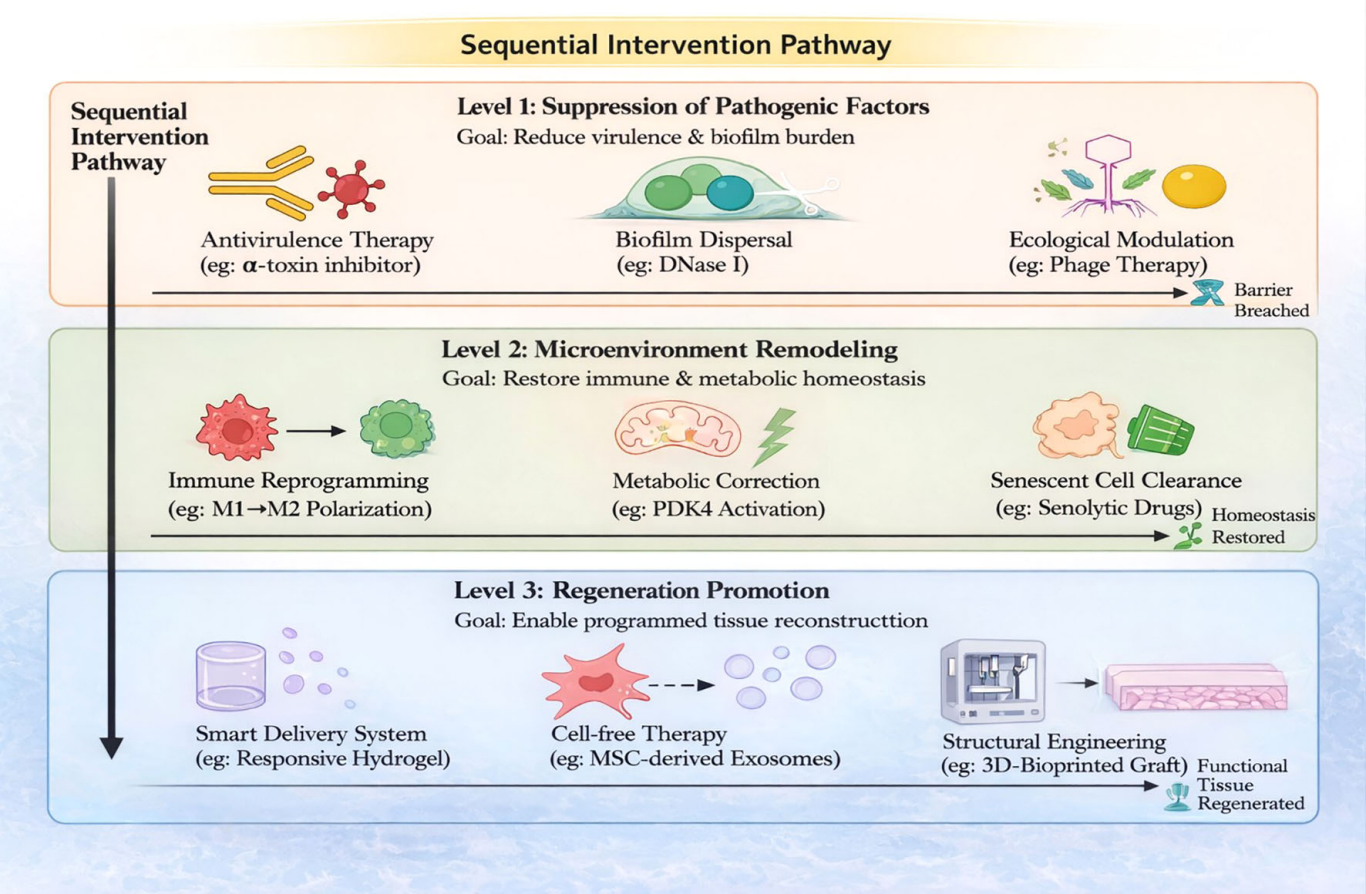
IDPPI-informed precision intervention framework for sequential reactivation of proliferative repair. Schematic overview illustrating a three-level, integrated therapeutic strategy derived from the IDPPI framework and governed by sequential, condition-dependent progression. Level 1 (Disarmament): The initial objective is to rapidly attenuate microbial pathogenic activity—rather than achieve non-selective eradication—through targeted antivirulence therapies, biofilm-dispersing agents, and targeted ecological modulation, thereby breaching pathogenic barrier modules (PBMs) and reducing ongoing injury to repair cells. Level 2 (Restoration): Following effective reduction of pathogenic pressure, therapeutic focus shifts toward remodeling the wound microenvironment, including immune reprogramming (e.g., M1-to-M2 macrophage polarization), correction of metabolic dysfunction, and clearance of senescent cells to restore immune–metabolic homeostasis and enable phase transition. Level 3 (Regeneration): Once a permissive microenvironment is established, spatiotemporally controlled tissue reconstruction can be achieved using smart delivery systems, cell-free regenerative therapies (e.g., extracellular vesicles), and tissue engineering approaches to promote functional tissue regeneration. Diamond-shaped decision nodes between levels indicate that advancement to subsequent therapeutic stages is contingent upon achieving predefined biological objectives of the preceding stage, enabling sequential reactivation of host proliferative repair rather than empirical escalation of antimicrobial therapy.

Conventional chronic wound management has long recognized that durable closure depends on more than antimicrobial escalation alone, emphasizing wound-bed preparation, barrier removal, and timing of advanced therapies (e.g., WBP/TIME and wound-infection continuum concepts) (45, 46). Consistent with this view, microscopy- and model-based studies report that biofilm-associated community states are enriched in chronic non-healing wounds and can impair clearance and repair permissiveness (6, 11). Clinically, even when microbial burden is partially reduced, wounds may remain stalled, suggesting that the host proliferative repair program can stay functionally constrained by persistent inflammatory, metabolic, and microenvironmental barriers (6, 22). Based on the IDPPI framework (defined in the Introduction), the following section outlines a stage−ordered therapeutic strategy that is complementary to standard−of−care (**Figure 4**).

Relationship to prior integrated paradigms. IDPPI aligns with wound−bed preparation/TIME, wound−infection continuum concepts (46, 47). and biofilm consensus guidance (32). Its distinct focus is treating persistent microbial pathogenic activity as a driver of proliferative execution failure, operationalizing “microbial damage potential” as measurable functions, and linking these to a closed−loop, biomarker−informed decision workflow across Disarmament → Restoration → Regeneration.

Building on this evidence, IDPPI reframes therapy from “pathogen presence” to microbial damage potential, measurable microbial functions that impede proliferative repair execution. Operationally, these functions include (1): virulence effector activity (toxins/proteases/secretion systems), (2) biofilm/PBM barrier functions (diffusional, metabolic, and immune obstruction), and (3) community-regulated programs that sustain these outputs (6). Because these functions are spatially and temporally heterogeneous and are not reliably captured by single snapshot measures, and because many function-targeted interventions remain supported primarily by preclinical or non–wound-specific clinical evidence, translation should be biomarker-aligned and cautious (46, 48).

Accordingly, IDPPI-guided therapy is conceptualized as a stage-ordered process: (1) Disarmament to suppress microbial damage potential (virulence outputs and biofilm/PBM maintenance), (2) Restoration to remodel immune–metabolic constraints that maintain proliferative arrest, and (3) Regeneration to deploy reconstruction-promoting approaches once a permissive microenvironment is re-established. This sequencing provides a mechanism-informed blueprint for selecting and timing interventions, aiming to reactivate proliferative repair rather than defaulting to empirical escalation of antimicrobials (6, 22).

### 3.1 Level 1 (Disarmament): functional suppression of virulence and biofilm programs

At the first therapeutic level, the goal is not necessarily complete microbial eradication, but rapid reduction of microbial damage potential by functionally suppressing dominant virulence outputs and biofilm/PBM barrier activity that perpetuate tissue injury and block proliferative repair. This “disarmament” logic is consistent with extensive evidence that biofilm-associated infections exhibit enhanced tolerance to antimicrobials and host clearance, and that virulence effectors can directly injure repair-competent keratinocytes, endothelial cells, and stromal cells, thereby compromising re-epithelialization and angiogenesis (6, 11, 49).

#### 3.1.1 Antivirulence targeting

In preclinical infection models, neutralizing *S. aureus* α-toxin (Hla) or inhibiting *P. aeruginosa* T3SS/ExoU reduces virulence-driven tissue injury (7–10). Importantly, the feasibility of clinical antivirulence biologics has been demonstrated in non-wound settings: the α-toxin–neutralizing mAb suvratoxumab (MEDI4893) was evaluated in a randomized phase 2 trial for prevention of *S. aureus* ventilator-associated pneumonia (SAATELLITE) (50), and the anti-Hla mAb AR-301 (tosatoxumab) has been studied as an adjunct to standard-of-care antibiotics in severe *S. aureus* pneumonia in ICU patients (51). For *P. aeruginosa*, T3SS-directed strategies include the anti-PcrV antibody fragment KB001-A (studied clinically in cystic fibrosis populations) (52), and the bispecific mAb gremubamab (MEDI3902) targeting PcrV and Psl, evaluated in mechanically ventilated ICU patients colonized with *P. aeruginosa* (EVADE) (53). In addition to antibodies, small-molecule antivirulence inhibitors provide proof-of-principle: the AgrA quorum-sensing inhibitor savirin attenuated *S. aureus* agr signaling and improved host defense in murine skin infection models (54), and the ExoU phospholipase inhibitor pseudolipasin A inhibits ExoU activity *in vitro* (55).

#### 3.1.2 Antibiofilm/PBM disruption

Biofilm-targeted interventions aim to destabilize the extracellular polymeric substance (EPS) matrix to weaken barrier functions and restore access for host defenses and antimicrobials. Enzymatic strategies include DNase I, dispersin B (PNAG hydrolase), and other glycoside hydrolases that degrade key EPS polysaccharides (56). In wound-relevant models, PslG/PelA glycoside hydrolases improved antibiotic penetration and potentiated clearance, including additive effects with tobramycin in *P. aeruginosa* wound infection (57). Broader screening in wound contexts further supports that selected GHs (e.g., α-amylase + cellulase) can enhance antibiotic-mediated clearance in mouse wound models and in diabetic settings (58).

At the same time, antibiofilm dispersal must be framed with appropriate safety constraints: large-scale *in vivo* dispersal of motile biofilm bacteria by GHs caused lethal septicemia in the absence of antibiotic therapy in a mouse wound model, indicating that dispersal strategies should be deployed prudently (e.g., paired with antimicrobials, controlled dosing/delivery, and careful patient selection) (48). Clinically, biofilm disruption is therefore most defensible as part of an integrated bundle with debridement, localized anti-infective measures, and downstream restoration/regeneration steps rather than as a stand-alone maneuver.

#### 3.1.3 Precision ecological modulation

Precision ecological modulation uses narrow-spectrum bacteriophages, engineered probiotics/consortia, or other targeted approaches to selectively suppress dominant pathogens or reshape community structure toward a more repair-permissive ecosystem (6, 59, 60). Early clinical evidence in the wound space is emerging: a randomized double-blind phase I/IIa study evaluated topical TP-102, a bacteriophage cocktail targeting *S. aureus*, *P. aeruginosa*, and *A. baumannii* in infected and non-infected diabetic foot ulcers, reporting safety/tolerability and microbiologic reduction signals (underpowered for efficacy) (60). A key conceptual advantage of ecological modulation is the potential to discriminate between pathogenic and beneficial community members (or functions), reducing collateral damage relative to indiscriminate eradication. In practice, however, target selection and delivery in polymicrobial biofilms remain major translational challenges, and ecological approaches should be presented as adjunctive, mechanism-guided options rather than universal replacements for standard wound care (32). Beyond phage cocktails, “selective” strategies can also operate at the function level rather than indiscriminate killing (61). For example, quorum-quenching approaches aim to suppress virulence coordination and biofilm maintenance with reduced collateral disruption to commensals, aligning with the goal of sparing beneficial community members while disabling pathogenic programs. In parallel, phage-delivered CRISPR antimicrobials and related precision constructs provide a route to selectively target resistance/virulence determinants or defined pathogenic strains, conceptually enabling microbiome editing rather than broad eradication (62). Finally, engineered probiotics/live biotherapeutics represent a complementary ecological route: they can competitively reshape community structure while providing pro-repair immune or epithelial cues, supporting repair-permissive states when deployed under appropriate safety and containment constraints (59).

Collectively, antivirulence, antibiofilm/PBM-directed, and ecological interventions can cooperate to dismantle coordinated pathogenic programs, reduce ongoing cellular injury, and create the conditions required for immune–metabolic restoration at subsequent stages of IDPPI-guided therapy. Major Level 1 strategies are summarized in **Table 5**.

**Table 5:** Antivirulence and antibiofilm strategies under investigation.

Strategy category	Primary target	Functional objective	Role within the IDPPI-guided therapeutic sequence
Antivirulence approaches	Pathogen virulence expression	Reduce direct injury to proliferative repair programs	Initial intervention to relieve proliferative inhibition
Antibiofilm strategies	Biofilm stability and burden	Destabilize persistent pathogenic niches	Restore accessibility to host regulation
Combined functional targeting	Virulence and biofilm	Break pathogenic steady state	Enable recovery of proliferative competence
Function-oriented modulation	Pathogenic activity rather than eradication	Minimize recurrence of impairment	Sustain progression into the proliferative phase

### 3.2 Level 2 (Restoration): immune–metabolic reprogramming to enable proliferative phase transition

Following partial control of virulence-driven injury, many chronic wounds remain stalled because the wound bed is still dominated by non-resolving inflammation, metabolic stress, and redox imbalance, which together sustain proliferative arrest and blunt fibroblast/keratinocyte/endothelial cell function. Experimental and clinical literature on diabetic and other chronic wounds consistently supports impaired transition from inflammatory to pro-repair immune states (e.g., persistent pro-inflammatory macrophage tone and elevated cytokines such as TNF-α), accompanied by mitochondrial dysfunction and oxidative stress that compromise matrix deposition and angiogenesis (63–65).

Immune remodeling and resolution. Rather than broad immunosuppression, restoration strategies aim to promote resolution of chronic inflammation and reprogram innate immune responses toward a repair-permissive state. Specialized pro-resolving mediators provide direct evidence for this approach: in obese-diabetic (db/db) mice, impaired endogenous pro-resolving lipid mediator signatures were observed in wounds, and local application of Resolvin D1 (RvD1) accelerated wound closure with reduced accumulation of apoptotic cells/macrophages and improved resolution dynamics (64, 66). Targeted cytokine modulation is supported primarily by preclinical evidence in wound-relevant models. For example, in a bacteria-inoculated wound model in type 2 diabetic (db/db) mice, TNF-α blockade with etanercept reduced apoptosis of matrix-producing cells and increased fibroblast number and new matrix formation during healing (67). More recently, comparative *in vivo* data in an impaired wound-healing mouse model indicate that different TNF inhibitors can exhibit distinct efficacy signatures in accelerating closure, underscoring that “TNF-α inhibition” is not mechanistically interchangeable across agents (68). Importantly, immune modulation must be coordinated with infection control; when microbial damage potential remains high, excessive immunosuppression may increase risk of invasive infection or delayed clearance. This stage therefore fits best after Level 1 disarmament has meaningfully reduced pathogenic pressure.

Metabolic correction and redox normalization. Metabolic restoration is particularly relevant in diabetes-associated chronic wounds, where hyperglycemia-driven mitochondrial stress and ROS burden undermine repair-cell bioenergetics. Mechanistic evidence supports targeting these constraints: in STZ-induced diabetic mice and human dermal fibroblasts, PDK4 insufficiency was linked to persistent senescence markers, whereas PDK4 overexpression improved the senescent phenotype and accelerated closure, with effects attributed to metabolic reprogramming (enhanced glycolysis) and reduced ROS, involving YAP/JNK signaling (69). Beyond fibroblast-centered interventions, emerging immunometabolic studies indicate that amino-acid pathways can tune inflammatory resolution during skin repair. For example, recent work reported that glutamine metabolism is enriched in macrophages during resolution and can suppress neutrophil recruitment to facilitate inflammatory termination and tissue repair (70–72). Translationally, these approaches should be integrated with systemic disease management and balanced between local versus systemic delivery.

Targeting senescence-like proliferative blockade (with window/safety caveats). Senescence and senescence-associated secretory phenotype (SASP) can reinforce chronic inflammatory tone and proliferative arrest in non-healing wounds, motivating senotherapeutic exploration. Senolytic strategies include FOXO4–p53 axis disruption: the senolytic peptide FOXO4-DRI was shown to selectively induce apoptosis of senescent cells and restore tissue homeostasis in preclinical models of chemotoxicity/aging (73, 74). However, senescence is context-dependent and can also contribute to normal repair programs; therefore, the timing, targeting specificity, and safety of senolytics in chronic wounds remain incompletely defined, and wound-specific validation is needed before clinical translation (75, 76).

Collectively, immune–metabolic reprogramming aims to remove microenvironmental constraints on cell proliferation and enable phase transition into effective granulation, angiogenesis, and re-epithelialization. Biomarker dimensions and mechanism-informed criteria for stratifying immune–metabolic imbalance and proliferative arrest at this stage are summarized in **Table 6**.

**Table 6:** Mechanism-informed stratification of infection-driven proliferative impairment.

Biomarker dimension	Representative indicators	Stage of clinical application	Pathophysiological implication	Guidance for stage-specific intervention
Microbial damage potential	Biofilm burden, virulence expression	Tier 1 (point-of-care/research)	Persistent functional injury	Prioritize antivirulence and antibiofilm control
Immune–metabolic status	Inflammatory and metabolic markers	Tier 0 (routine) + Tier 1 (adjunct)	Inflammation–proliferation uncoupling	Initiate immunometabolic remodeling
Regenerative capacity	Cell-cycle arrest markers	Tier 2 (exploratory/research)	Suppressed execution of the proliferative program	Introduce regenerative support after control

### 3.3 Level 3 (Regeneration): execution of programmed tissue reconstruction under permissive microenvironments

Only after microbial pathogenic pressure has been meaningfully attenuated and immune–metabolic constraints have been relaxed can regeneration-promoting strategies be expected to perform reproducibly. At this stage, advanced biomaterials and regenerative technologies function as precision amplifiers of repair, improving spatiotemporal coordination of angiogenesis, matrix remodeling, and re-epithelialization, rather than attempting to override an actively hostile wound microenvironment.

Responsive smart delivery systems. Stimulus-responsive platforms (e.g., hydrogels and nanoparticles) can sense wound-associated cues such as pH, protease activity, and oxidative stress, enabling localized and time-matched release of growth factors, extracellular vesicles, or small molecules to align therapy with dynamic wound biology (77–79). A representative example is an MMP-9–responsive hydrogel designed for diabetic wounds, reported to promote healing by suppressing endothelial-cell ferroptosis, thereby supporting vascular repair processes central to successful tissue reconstruction (80). For translation, key design requirements include (1) selecting triggers that are demonstrably enriched in the target wound state (e.g., elevated protease activity), (2) ensuring biocompatibility and predictable degradation, and (3) defining release kinetics that match the intended repair window rather than maximizing payload (77, 78, 80).

Cell-free regenerative therapies (MSC-/endothelial cell–derived exosomes). Cell-free strategies increasingly rely on extracellular vesicles/exosomes as carriers of pro-repair microRNAs and proteins that can modulate inflammation and promote angiogenesis without the logistical and safety complexities of live-cell transplantation (81–83). In diabetic wound models, exosome-based treatments have been reported to accelerate closure while enhancing endothelial function and promoting macrophage polarization toward repair-associated phenotypes, for instance, hypoxia-conditioned endothelial cell–derived exosomes were shown to facilitate diabetic wound healing by improving endothelial performance and supporting M2 polarization (84). However, the translational bottleneck is often not conceptual efficacy but manufacturability and standardization: donor/source variability, upstream cell culture conditions, isolation/purification consistency, batch-to-batch reproducibility, stability during storage/transport, and mechanism-linked potency assays (e.g., angiogenic activity and immunomodulatory readouts) are frequently limiting steps for clinical-grade deployment (81–83, 85). These issues should be stated explicitly rather than summarized only as “quality control remains a barrier.”

Structural tissue engineering and 3D bioprinting. Structural approaches, including 3D printing/bioprinting, aim to fabricate skin substitutes with spatially organized cells, bioinks, and bioactive components, providing architecture-informed solutions for complex or full-thickness defects (86–88). Reviews highlight expanding capabilities such as integrating stem-cell delivery, antimicrobial strategies, and sensor-enabled constructs to better match the needs of chronic wound environments (86, 88). Despite promise, persistent challenges remain: achieving rapid and durable vascular integration, maintaining immune compatibility in compromised hosts, and ensuring long-term mechanical stability and functionality, particularly when wounds have a history of infection or metabolic impairment (86, 87).

Together, Level 3 interventions operationalize programmed reconstruction once upstream constraints have been addressed, enabling coordinated tissue repair (vascularization–matrix–epithelium coupling) rather than isolated growth stimulation.

### 3.4 Translational closed loop: biomarker-driven adaptive precision therapy

Operational definition of IDPPI−positive status. We propose that a chronic wound can be operationally classified as IDPPI−positive when both of the following criteria are met: (1) evidence of sustained microbial damage potential (e.g., bedside biofilm signal ≥2+ on a validated assay, or detection of virulence−associated factors such as S. aureus α−toxin or P. aeruginosa T3SS effectors), AND (2) functional impairment of proliferative repair execution (e.g., failure to progress to granulation or epithelialization after ≥4 weeks of standard care, or elevated cell−cycle arrest markers such as p21/p27 in wound tissue). This operational phenotype is intended as a translational stratification tool, not a validated diagnostic score, and its clinical utility requires prospective validation.

**Figure 5:**
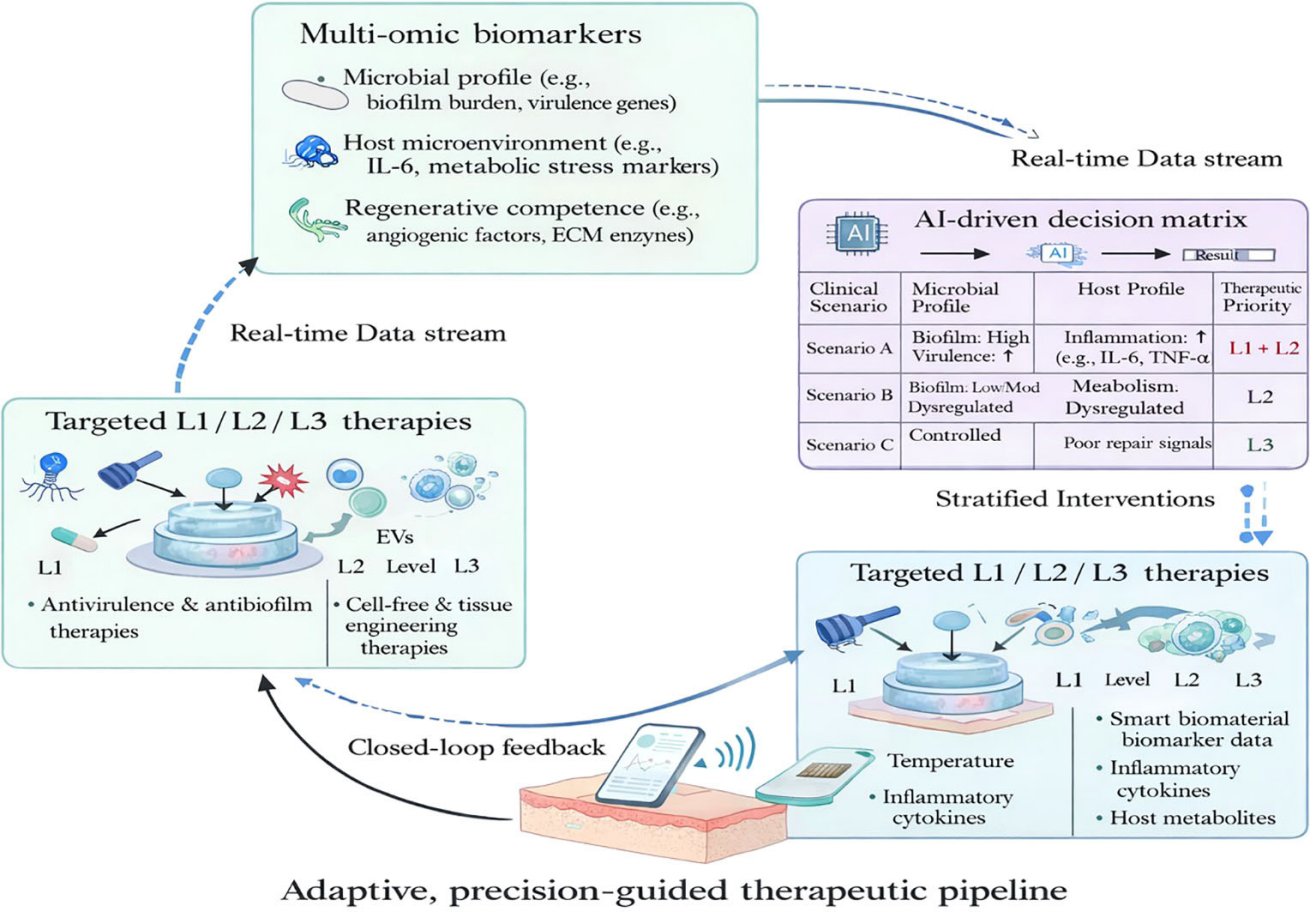
Biomarker-guided adaptive decision matrix for dynamic precision wound management. This schematic illustrates translation of the IDPPI framework into a feedback-guided workflow, data-driven system for dynamic precision wound management. (Center panel) Real-time monitoring of multidimensional biomarkers spanning three mechanistic domains—microbial pathogenic activity, host immune–metabolic microenvironment, and tissue regenerative capacity—provides continuous input streams for wound state assessment. (Right panel) Artificial intelligence integrates these data to stratify wounds into distinct functional scenarios (e.g., Scenario A: high pathogenic activity with active inflammation; Scenario B: persistent inflammation with metabolic dysregulation; Scenario C: controlled infection but insufficient regenerative signaling) using a decision matrix, thereby generating prioritized therapeutic recommendations corresponding to Level 1 (Disarmament), Level 2 (Restoration), or Level 3 (Regeneration). (Left panel) These outputs activate level-specific intervention modules from a stratified therapeutic repertoire, enabling targeted suppression of pathogenic programs, microenvironmental remodeling, or regenerative execution as appropriate. The feedback pathway depicted at the bottom indicates continuous post-intervention reassessment of wound status, allowing iterative refinement of therapeutic strategies and enabling sequential reactivation of host proliferative repair through adaptive, precision-guided management.

Effective implementation of the three-level IDPPI-guided strategy requires a feedback-guided measure → interpret → intervene → re-measure loop (operationalized in Section 3.5), rather than one-time staging (**Figure 5**). Standard bedside assessment may capture gross wound appearance but often under-resolves temporal changes in biofilm-associated barrier burden, pathogen/strain-level risk signals, and host permissiveness for proliferative repair. This can result in premature escalation to regenerative therapies or prolonged empiric antimicrobial intensification. A mechanism-aligned biomarker framework enables longitudinal identification of the dominant bottleneck at each reassessment and supports stepwise (and reversible) decisions across Disarmament → Restoration → Regeneration (32, 89–102).

#### 3.4.1 Measure: tiered biomarkers that fit real-world workflows

To preserve feasibility, IDPPI measurement can be implemented in tiers, escalating only when additional resolution is likely to change management:

Tier 0 (routine bedside/standard-of-care, every visit). Track wound area trajectory and granulation/epithelialization kinetics, exudate characteristics, perfusion surrogates, offloading adequacy, and systemic control (e.g., glycemia). These serial trends provide the minimum dataset for the closed loop (Section 3.5).

Tier 1 (point-of-care/accessible assays when clinically indicated).

Bedside biofilm detection and barrier surrogates: point-of-care wound blotting/visual grading has been evaluated against fluorescence imaging and linked to healing outcomes, supporting practical identification of biofilm-dominant states (89). Broader bedside-accessible biofilm assessment options and their limitations are summarized in recent diagnostics-focused work (90). Biofilm identification and treatment should remain consistent with consensus guidance for chronic nonhealing wounds (32).

Pathogen-targeted POC sensing: electrochemical detection of Pseudomonas in wound exudate illustrates feasible pathogen-directed sensing in chronic wound settings (91).

Volatilomics/VOC sensing and electronic noses (e-noses): rapid functional readouts of microbial activity.

Wound pathobiomes generate volatile organic compounds (VOCs) as metabolism-linked outputs that can encode species- and community-level signatures. Electronic noses (e-noses), sensor arrays coupled with pattern-recognition models, have been proposed as rapid, non-invasive tools to support wound infection screening and longitudinal monitoring, offering workflow-compatible signals when culture-independent profiling is not feasible at the bedside. Current reviews emphasize feasibility and speed but also note that robust clinical validation remains limited; sampling interfaces (headspace control under dressings), humidity/exudate confounding, and model generalizability across wound types are key translational constraints (103).

Proof-of-principle work in diabetic foot infection contexts further suggests discriminatory capacity: headspace volatile sensing benchmarked against GC–MS validation reported that multivariate/machine-learning classifiers could differentiate single versus polymicrobial patterns with high accuracy in the reported setting, supporting VOC sensing as a potential Tier 1 “fast signal” that can trigger timely re-assessment and confirmatory testing rather than replacing guideline-based infection diagnosis (104).

Systemic infection/inflammation adjuncts (risk context, not wound-specific substitutes): PCT has evidence as an infection-associated biomarker in DFU infections (meta-analysis) (92), and inflammatory cytokines (including IL-6) have been evaluated as part of infection assessment in venous leg ulcers (93). These markers should be interpreted as adjuncts that contextualize risk (e.g., possible systemic involvement), while diagnosis and antimicrobial intensity should follow established DFI criteria and guideline frameworks (94).

Tier 2 (selected refractory/high-risk cases).

Strain-/species-resolution profiling: strain- and species-level variation in diabetic wound microbiomes is associated with clinical outcomes and therapeutic efficacy, supporting the concept that “which strain dominates” can matter for prognosis and for tailoring strategies beyond culture-only approaches (95). Comparative studies of host–bacterial interplay across ulcer types further reinforce the value of integrating microbial features with host context (96).

Mechanistic immunometabolic context (translational bridge): immune signaling can rewire macrophage metabolism via the citrate/ACLY axis to shape epigenetic programs, providing mechanistic rationale for metabolite-informed panels while recognizing that clinic-ready local metabolite assays remain an evolving translational area (97).

Proliferative blockade/senescence burden: senescence-linked proliferative arrest programs (including p21-associated pathways) influence cutaneous wound repair dynamics and motivate targeted assessment in non-healing phenotypes (98, 99).

Protease burden as a regenerative barrier: elevated MMP-9 predicts poor DFU healing and can serve as a practical biochemical indicator that the wound bed remains protease-dominant and matrix-unstable, conditions that are likely to blunt downstream regenerative inputs (100).

Baseline risk framing (not for rapid closed-loop monitoring): host genetic susceptibility has been synthesized for DFU risk but is less suited for short-interval adaptive decisions (101).

Implementation principle: closed-loop decisions should prioritize within-patient trajectories (serial change) over single thresholds, especially for bedside biofilm indicators, inflammatory surrogates, and protease signals (89, 90, 92, 100).

#### 3.4.2 Interpret → intervene: a decision matrix for stratification and sequential therapy

Interpretation should identify the dominant failure mode at each reassessment (often mixed), then select the corresponding level(s) and actions:

1. Biofilm-/barrier-dominant pattern → Level 1 (Disarmament) priority.

Positive/strong bedside biofilm signals and/or clinical patterns consistent with biofilm-associated stagnation support prioritizing debridement and biofilm-directed measures before escalating regenerative inputs, in line with chronic wound biofilm consensus guidance (32, 89, 90). Pathogen-targeted cues (e.g., Pseudomonas detection) can further justify refining antimicrobial selection rather than default broad-spectrum escalation (91, 94).

Suspected invasive infection/systemic inflammatory risk context → hold/strengthen Level 1 and avoid premature immunomodulation.

When adjunct systemic biomarkers (e.g., PCT) suggest higher infection-associated burden, interpretation should emphasize alignment with guideline diagnostic criteria and treatment recommendations for diabetes-related foot infections rather than reflex intensification or premature transition to restoration/regeneration (93, 94). Cytokine panels may provide supportive inflammatory context but should not substitute for DFI diagnostic frameworks (93, 94).

Persistent microbial risk despite partial control → refine targeting and reassess dominance.

Where available, strain/species-level data linked to outcomes and therapeutic response can support refinement of targeting and reassessment of dominant community structures, especially in refractory DFU phenotypes (95, 96).

2. Protease-dominant, matrix-unstable bed → Level 2 (Restoration) gating before Level 3.

Persistently elevated protease burden (e.g., MMP-9 associated with poor DFU healing) indicates a non-permissive bed for durable reconstruction; prioritize microenvironment restoration and stabilization before expecting consistent benefit from regenerative scaffolds/skin substitutes (100).

3. Regeneration-ready pattern → conditional Level 3 (Regeneration) as a timed amplifier.

When biofilm/infection pressure is controlled per guideline-aligned criteria and the wound demonstrates improving permissiveness (reduced barrier/protease dominance with renewed proliferative kinetics), advanced biomaterials and topical regenerative options can be introduced as conditioned amplifiers to accelerate closure and improve durability. Comparative evidence syntheses for DFU biomaterials/topical medications support this escalation concept while underscoring heterogeneity and the need for appropriate patient selection and timing (102).

Step-back rule (core closed-loop behavior): if Level 3 is initiated but subsequent reassessment shows re-emergent biofilm/barrier dominance or renewed protease instability, the framework explicitly supports stepping back to Level 1/2 until permissiveness is re-established (32, 89, 90, 100).

#### 3.4.3 Re-measure: shortening feedback latency with real-time and rapid diagnostics

Closed-loop performance improves when state transitions are detected earlier than routine clinic intervals. A microfluidic wearable platform for wound exudate management and analysis demonstrates the feasibility of near-continuous monitoring in human chronic wounds (105). For microbiology, combining CRISPR-Cas12a detection with metagenomic sequencing provides a paradigm for rapid and broad microbial detection in infectious diabetic foot samples, supporting faster alignment of therapy with actual microbial states and resistance determinants (106). Together, these tools can reduce delay between state change and treatment adjustment, improving timing of escalation and de-escalation across IDPPI levels (105, 106).

### 3.5 Clinical implementation: workflow, target populations, and an illustrative DFU case

#### 3.5.1 Clinical implementation: complementary to standard-of-care rather than a replacement

Clinical implementation of IDPPI is intended to complement—not replace—standard wound care. Foundational measures (debridement, offloading/pressure redistribution, perfusion optimization, metabolic control, and guideline-aligned infection evaluation/management) remain first-line. IDPPI primarily informs how to sequence adjunctive interventions when healing remains stalled: functional disarmament of pathogenic activity and PBM/barrier burden (Disarmament), restoration of a repair-permissive immune–metabolic microenvironment (Restoration), and only then regenerative enablement (Regeneration). Consistent with guideline principles, the framework does not advocate indiscriminate microbial eradication; instead, it motivates function-targeted suppression and stepwise escalation/de-escalation based on longitudinal reassessment.

#### 3.5.2 Illustrative DFU workflow example

Clinical implementation (Section 3.5) operationalizes the closed-loop workflow defined in Section 3.4 by specifying feasible clinic-facing steps (visit cadence, triggers for escalation/de-escalation, and target populations) and by illustrating how Disarmament → Restoration → Regeneration decisions can be executed in a DFU case while maintaining standard-of-care fundamentals (debridement, offloading, perfusion optimization, and guideline-aligned infection management). Re-measure: reassess at clinically appropriate intervals using predefined response triggers (e.g., improved granulation/epithelialization kinetics, reduced exudate/inflammatory features, improved bedside biofilm signals) to calibrate antimicrobial intensity and to introduce regeneration-promoting therapies only after a permissive microenvironment is reasonably established.

In a DFU that fails to progress despite optimal offloading, debridement, and perfusion optimization, closed-loop reassessment may indicate persistent pathogenic activity (e.g., recurrent biofilm/PBM features and barrier-associated injury signals) even when overt infection signs are reduced. Rather than prolonged empiric broad-spectrum antimicrobial intensification, the IDPPI workflow would prioritize barrier disruption and function-targeted disarmament, followed by reassessment of repair permissiveness (granulation quality, epithelial edge advancement, and inflammation/metabolic control). Once pathogenic activity is functionally suppressed and restoration targets are met, advanced regenerative therapies can be deployed with a higher likelihood of durable response. Persistent non-response would trigger re-interpretation and escalation to higher-resolution diagnostics in selected refractory cases.

The framework may be most immediately applicable to DFU, long-standing venous leg ulcers, pressure injuries, and other chronic wounds where recurrent infection signals, suspected biofilm burden, and proliferative stagnation coexist. Patients with repeated antibiotic exposure, recurrent wound breakdown, or a mismatch between partial infection control and persistent failure to granulate/epithelialize may represent high-yield candidates for IDPPI-guided stratification.

Illustratively, in a DFU with stalled granulation despite repeated empiric antibiotics, an IDPPI-guided reassessment may prioritize debridement and biofilm-directed measures consistent with biofilm consensus guidance (32), while aligning antimicrobial escalation with established diagnostic/severity criteria for diabetes-related foot infection rather than default broad-spectrum intensification (94). If serial reassessment shows reduced indicators of microbial damage potential and improving wound kinetics, a regenerative scaffold/skin substitute or other advanced biomaterials/topical options can then be introduced as a conditionally timed amplifier to accelerate closure and improve durability of repair (102). This aligns with comparative evidence syntheses in DFU. If the wound deteriorates or biofilm/infection signals re-emerge after escalation, the workflow explicitly supports holding or stepping back to Level 1/2 before continuing regenerative execution (32, 94).

Targeted molecular assays, when available, may further refine stratification in selected refractory cases. Taken together, the IDPPI framework complements rather than supplants current clinical guidelines, and its adoption should be phased alongside standard wound−bed preparation and infection management.

## 4 Future directions: toward intelligent, adaptive, and precision-guided wound management

A deeper understanding of IDPPI not only informs current therapeutic strategies but also defines a developmental trajectory for next-generation chronic wound management. Because this pathological state emerges from dynamic interactions among microbial pathogenic programs, host immune–metabolic responses, and regenerative execution, future advances will require integrative, multidisciplinary frameworks. Systems biology, biomimetic modeling, artificial intelligence–assisted monitoring, and responsive biomaterials are expected to converge toward intelligent, adjustable therapeutic systems capable of dynamically restoring proliferative repair.

### 4.1 Decoding complexity: from multi-omics data to mechanistic networks

Chronic wounds represent dynamic host–microbe interaction systems rather than static pathological entities. Future research will increasingly rely on spatially resolved multi-omics integration to capture this complexity. By combining spatial transcriptomics, proteomics, and metabolomics, it becomes possible to map cellular composition, intercellular communication, and functional states at tissue scale. Single-cell transcriptomic studies have begun to delineate the cellular landscape of DFU, identifying fibroblast and immune cell subsets associated with impaired healing (107). When integrated with metabolomic profiling of the wound microenvironment, these datasets enable reconstruction of molecular circuits driving proliferative stagnation and reveal actionable intervention targets (108, 109).

In parallel, incorporation of spatial microbiome profiling and virulence expression mapping links microbial community architecture with host responses within a unified coordinate system. Such integrative analyses are essential for building interpretable mechanism-informed network models that reflect the hierarchical processes underlying IDPPI.

### 4.2 Human-relevant models: from animal studies to biomimetic systems

Although animal models have provided critical mechanistic insights, they incompletely recapitulate human chronic wounds, particularly with respect to polymicrobial ecology and disease-specific microenvironments. Emerging biomimetic platforms, including organ-on-a-chip and organoid systems, offer experimental models that more closely approximate human physiology. Microfluidic technologies enable reconstruction of dynamic cellular interactions relevant to wound healing, such as bidirectional macrophage–fibroblast signaling under controlled flow and gradient conditions (110). Tissue-specific platforms, including gingiva-on-a-chip systems, have already been applied to study host responses to implanted materials (111).

By enabling controlled co-culture of microbial communities with host cells under defined oxygen tension, glucose availability, and pH gradients, these platforms better mimic the hostile microenvironment characteristic of chronic wounds. Such biomimetic systems hold promise for screening anti-infective materials, validating immunomodulatory strategies, and dissecting host–microbe interactions, thereby accelerating translational development of candidate interventions (112).

### 4.3 Clinical decision support: integrating artificial intelligence and real-time sensing

As chronic wound care advances toward precision medicine, clinical decision-making increasingly depends on continuous data acquisition and intelligent interpretation. Artificial intelligence (AI) and machine learning have demonstrated promising performance in wound image analysis and healing outcome prediction (113). Standardized validation frameworks are now being developed to assess clinical reliability and generalizability of image-based tools, facilitating their integration into routine practice (114, 115).

Parallel advances in wearable sensing technologies enable real-time monitoring of wound physiology. Flexible, multi-parameter sensors continuously measure temperature, pH, moisture, and infection-related biomarkers, including inflammatory mediators and bacterial metabolites, via wireless transmission (116, 117). Battery-free and multi-channel platforms support long-term monitoring through skin-compatible patches (118). When integrated with AI-driven analytical pipelines, these data streams enable quantification of wound dynamics, early risk detection, and personalized adjustment of therapeutic strategies over time (105).

Future integration of VOC sensing into closed-loop wound care. An actionable next step is the integration of VOC sensing (including e-noses) into wearable or dressing-compatible platforms to shorten feedback latency. By coupling VOC pattern streams with AI-driven classification, such systems could provide early warnings of pathobiome reconfiguration before overt clinical deterioration, enabling earlier return to Level 1 disarmament or refinement of targeting. However, translation will require standardized sampling interfaces (headspace control under dressings), calibration across wound types and care settings, and prospective trials demonstrating added value beyond standard infection criteria and antimicrobial stewardship frameworks (103).

### 4.4 Toward feedback-guided workflow therapeutic systems with programmable delivery

A central future objective is to integrate multi-source biological data with advanced materials engineering. This would generate localized therapeutic systems that can sense, provide feedback, and deliver on-demand intervention. Such platforms enable precise alignment between therapeutic delivery and pathological signals, which is an essential requirement for restoring proliferative repair programs. For example, enzyme-responsive hydrogels releasing therapeutic agents in response to elevated MMP-9 levels protect endothelial cells and enhance angiogenesis in diabetic wounds (80). Dual-responsive materials sensitive to glucose and oxidative stress dynamically regulate metabolic and redox microenvironments, thereby promoting vascular regeneration (119).

Materials incorporating feedback-guided workflow control logic represent a paradigm shift from passive wound dressings toward programmable local delivery platforms (120, 121). Their clinical translation will depend on accurate trigger calibration, optimized release kinetics, and seamless integration into existing care workflows. Representative smart material–based strategies enabling programmed regeneration following control of IDPPI are summarized in **Table 7**.

**Table 7:** Programmed regeneration enabled after control of infection-driven impairment.

Functional role	Sensed wound signals	Triggered response	Contribution following control of IDPPI
Signal-responsive materials	Infection, inflammation, metabolic cues	On-demand release of therapeutics	Prevent premature or ineffective regenerative stimulation
Responsive delivery systems	Dynamic microenvironmental changes	Adaptive and stage-specific dosing	Enhance efficiency of the proliferative repair program
Regeneration-oriented dressings	Proliferative repair demand signals	Support cell proliferation and matrix remodeling	Improve quality and stability of proliferative regeneration
Monitoring-integrated platforms	Multidimensional biomarker dynamics	Closed-loop regulation	Enable programmed and sequential regeneration

## 5 Conclusions and future perspectives

Here, we propose IDPPI as an analytical framework that reframes chronic wound non−healing as a consequence of infection−driven proliferative repair failure. By explicitly linking microbial pathogenicity to proliferative execution arrest, IDPPI provides a mechanism−informed foundation for stratified and sequential therapeutic intervention, shifting management beyond non−selective antimicrobial control toward mechanism−guided, stage−specific targeted therapy.

Looking forward, advances in spatial multi-omics, biomimetic human-relevant models, real-time sensing technologies, and artificial intelligence are poised to transform this conceptual framework into clinically actionable systems. By enabling dynamic and quantifiable assessment of wound states and adaptive adjustment of therapeutic strategies, chronic wound care may evolve from static staging and empirical treatment toward feedback-guided workflow, targeted regenerative medicine (105, 107, 108, 110, 111, 113, 118).

Operational details of the feedback-guided (closed-loop) IDPPI workflow are described in Section 3.5.

We acknowledge that translating feedback-guided workflow IDPPI systems into routine care faces practical barriers, including cost and accessibility of high-dimensional profiling, requirements for sampling standardization and computational infrastructure, and the need for regulatory-grade clinical validation demonstrating added value beyond existing wound assessment and antimicrobial stewardship practices. A staged roadmap may accelerate translation: near-term adoption can emphasize workflow-compatible proxies and targeted assays integrated into standard care, while multicenter studies establish standardized panels, decision thresholds, and outcome-linked validation. Longer-term integration of spatial multi-omics, real-time sensing, and AI decision support should proceed in parallel with prospective trials, interoperability standards, and equity-focused implementation to avoid widening care disparities.

Collectively, IDPPI offers not only a unifying explanation for chronic non-healing but also a roadmap for future innovation in wound management, bridging mechanism-informed insight with translational implementation.
